# Statement by King Edward's Hospital Fund

**Published:** 1920-03-27

**Authors:** 


### PRESENT POSITION OF THE LONDON VOLUNTARY HOSPITALS.

### Statement by King Edward's Hospital Fund.

The Honorary Secretaries of King Edward's Hospital Fund have issued the following statement on the present position of the London Voluntary Hospitals.

---

As the facts that have already been published about the difficulties of individual hospitals might give the impression that it is useless to attempt to tide the Voluntary Hospitals over the present crisis, it seems desirable that the King's Fund should publish a general statement on the position of the London hospitals taken as a whole, and thus give a rough indication of the scale of the problem which has to be faced.

In order to make its annual distribution in December, the King's Fund studies the financial position of each institution as shown by the audited accounts of the last complete year. The figures collected for this purpose have the disadvantage that they refer to the past, while, on the other hand, they have the advantage that they are based on definite audited accounts. From the surpluses, deficits, and debts, shown by these figures, it is possible to make a summary which indicates, though only very roughly, the extent to which the general position of the hospitals has improved, or the reverse during a series of years.

At the beginning of the war it was anticipated that the hospitals would be faced with immediate financial difficulties from increased prices and decreased contributions, but, as a matter of fact, the difficulties were so far balanced by the additional activities and generosity set in motion by the war, that the actual crisis was postponed, and is only now being experienced.

What actually happened seems to have been somewhat as follows :—

1913 was, for the London hospitals as a whole, a good year. They therefore began the war period with funds in hand.

1914 was only a moderately good year, but money was saved by the general postponement of building schemes.

1915 was distinctly a bad year, and prices began to rise.

1916 was a fairly good year.

1917 was not so good, and

1918 showed little if any improvement, and, as is well known, since 1918 prices have continued to rise, and War Office payments for beds occupied by soldiers have ceased.

In view of the results of 1913 and 1914, the King's Fund decided not to distribute the money saved by the suspension of building schemes or received from large legacies or other exceptional windfalls, until it was known to what extent the hospitals would be affected and which hospitals would need help most. The amounts actually distributed followed the conditions of the previous year fairly closely, the figures being as follows : In 1913 the Fund distributed £157,500; 1914, £140,000; 1915, £140,000; 1916, £170,000; 1917, £190,000; 1918, £200,000; and 1919, £230,000.

The increased distribution in 1919 was due to the fact that the total was based not only on the figures for 1918, the last year of the war, but also partly on the anticipated post-war needs of the hospitals, especially the necessity of carrying out deferred renewals and repairs. To effect this increase the Fund had to draw to the extent of about £46,000 on the reserves created during the war by exceptional legacies.

It is not yet possible to collect complete figures for the year 1919, but so far as they are available they indicate that the London hospitals, taken as a whole, after setting off the surpluses against the deficits, ended the year with a net deficiency of rather more than £210,000. This is undoubtedly a large sum, but their total annual expenditure is rather more than £2,100,000, and this means that, although a great part of their expenses has been doubled, like those of everybody else, by the rise in prices, and still further increased by the development of expensive methods of treatment and by additions to the number of beds, they were still able to meet out of their income for the year, including the grants from the Central Funds, about 90 per cent. of the increased total. It would only have been necessary for the public to subscribe, either direct to the hospitals in proportion to their individual claims, or through the King's Fund, a further sum equal to the amount which that Fund distributed last year, and the aggregate income of the hospitals in 1919 would have met their expenses.

It is certain that the expenditure of hospitals has increased since the end of 1919, and will continue to do so. Whether their income will increase or not depends largely on whether the loss of contributions from those whose incomes have fallen off during the war is balanced by contributions from those whose incomes have increased. It is neither desirable nor possible for the King's Fund to finance the whole work of the hospitals. But if the efforts now being made to tap new sources of revenue are actively pushed forward and give promise of future success, the King's Fund, if it is itself supported by the public, can do much in helping to secure that success.

It can do this by maintaining a steady and, if possible, gradually increasing distribution, or by giving exceptional assistance in times of exceptional difficulty. If sufficiently supported it could even do both. The hospitals of London, with their endowments, their subscription lists, and their other sources of revenue, backed by the King's Fund, the Metropolitan Hospital Sunday Fund, and the Hospital Saturday Fund, have a powerful momentum. They have to be tided over a period of difficulty, and if the public once realise what an important part the voluntary hospitals play in the medical service of the country and how much of their special value depends on the fact that they are voluntary hospitals, co-operating with other agencies but filling a place which no other agency can fill, there is no reason to fear that the present difficulties cannot, if sufficiently energetic steps are taken, be successfully surmounted.

On the question of the need for further hospital provision, the King's Fund has before it particulars of extension schemes submitted by a large number of London hospitals. Already, since 1913, more than 1,000 beds have been added, and no doubt account for part of the deficit mentioned above. The further schemes proposed would add another 3,000 beds, and would involve the expenditure of a very large sum for building and a large increase in maintenance charges. Moreover, numerous schemes of improvement not involving additional beds have been submitted. Towards this capital expenditure the King's Fund is about to allocate, in addition to a share in the ordinary distribution later on, the sum of £250,000 surplus Red Cross funds entrusted to it for this special purpose. But this will not go very far, and obviously these numerous schemes require careful examination, especially the larger ones, in order to ensure that only schemes that are financially practicable are undertaken, and that the money available for building is expended only where it will produce the greatest benefit.

These facts as to the general position of the London hospitals, taken as a whole, will serve to indicate the scale of the problem. The present system, in London alone, provided last year nearly £2,000,000 towards a total expenditure of less than £2,200,000. The difference between expenditure and income may well be greater this year. The difficulties will not be solved without a resolute effort, but there is no reason to consider them insoluble.

